# Seroprevalence of African horse sickness in selected donkey populations in Namibia

**DOI:** 10.14202/vetworld.2020.1005-1009

**Published:** 2020-05-31

**Authors:** Umberto Molini, Guendalina Zaccaria, Erick Kandiwa, Borden Mushonga, Siegfried Khaiseb, Charles Ntahonshikira, Bernard Chiwome, Ian Baines, Oscar Madzingira, Giovanni Savini, Nicola D’Alterio

**Affiliations:** 1 Department of Pathobiology, School of Veterinary Medicine, Faculty of Agriculture and Natural Resources, University of Namibia, Neudamm Campus, Namibia; 2 Istituto Zooprofilattico Sperimentale dell’Abruzzo e del Molise “G. Caporale” 64100 Teramo Italy; 3 Department of Virology, Central Veterinary Laboratory, 24 Goethe Street, Windhoek, Namibia

**Keywords:** African horse sickness, donkeys, enzyme-linked immunosorbent assay, Namibia

## Abstract

**Background and Aim:**

African horse sickness (AHS) is a non-contagious viral disease of horses and other equids caused by an arbovirus belonging to the *Reoviridae* family and genus *Orbivirus*. AHS is an endemic disease that is responsible for the death of a high number of horses every year in Namibia. At present, there is no information on the prevalence and distribution of AHS virus (AHSV) serotypes in the different regions of Namibia. Therefore, this survey aimed to fill this knowledge gap by investigating the AHSV seroprevalence in Namibian donkeys.

**Materials and Methods:**

A total of 260 blood samples (20 samples for each region) were randomly collected from donkeys aged between 3 and 5 years. Sera were screened for AHSV-specific immunoglobulin G antibodies using acommercial competitive enzyme-linked immunosorbent assay kit and samples positive to AHSV antibodies were further tested by serum neutralization (SN) assay to evaluate the AHSV serotype-specific immune response.

**Results:**

Seroprevalence of antibodies against AHSV in Namibian donkeys was 63.5%. The AHSV prevalence was significantly higher in the northern region (64%) than in the southern region (36%). A significantly (p<0.05) higher number of donkeys had antibodies against AHSV-6 (37.8%) and AHSV-9 (37.8%). The AHSV-2, AHSV-6, and AHSV-9 prevalence were higher (p<0.05) in the northern regions compared to the southern regions. None of the donkeys in this study, however, tested positive for AHSV-8.

**Conclusion:**

Results of the current study indicate that all AHSV serotypes have either circulated previously or are circulating in Namibia except for AHSV-8. In particular, AHSV-1, -2, -3, -4, -5, -6, and -9 serotypes have circulated or are circulating in the northern region of Namibia, while AHSV-1, -4, -5, -6, -7, and -9 have infected donkeys in the south. AHSV-9 and AHSV-6 were the most prevalent serotypes detected in donkeys in this study. SN results showed that several donkeys from Kavango East, Kavango West, and Ohangwena regions had been exposed to multiple serotypes, indicating the possibility of cocirculation of several strains in Namibia.

## Introduction

African horse sickness (AHS) is a non-contagious viral disease of horses and other equids (mules, donkeys, hinnies, and zebras) caused by an arbovirus belonging to the *Reoviridae* family, genus *Orbivirus*, and transmitted by hematophagous *Culicoides* midges [1]. The virus can cause an acute or subacute fatal disease in unvaccinated horses, characterized by respiratory and/or circulatory impairments [2]. In African donkeys (*Equus africanus*) and zebra (*Equus quagga*), infection is generally asymptomatic and the two species are considered to be vertebrate reservoirs of the virus [2]. To date, nine distinct serotypes of AHS virus (AHSV) have been identified by serum neutralization (SN) and molecular-based assays [3]. AHS is endemic in sub-Saharan Africa – from Senegal in the west to Ethiopia and Somalia in the east and extending southward down the African Atlantic seaboard to as far south as South Africa, with sporadic escapades into North Africa, the Middle East, and Mediterranean countries [1,4].

AHS is an endemic disease that is responsible for the death of a high number of horses every year in Namibia. Vaccination is the most effective measure to protect animals, reduce losses associated with the disease, prevent transmission to vectors, and, eventually, allow the eradication of the disease. Live-attenuated vaccines for use in horses, mules, hinnies, and donkeys are currently available. Vaccination with live-attenuated strains of AHSV is the primary means of controlling AHS in endemic areas. Concerns have been raised regarding the use of live-attenuated vaccines due to their ability to revert to virulence, their potential for reassortment with field AHSV strains, transmission by vectors, and the difficulty of differentiating between infected and vaccinated animals [5,6]. Due to their resistance to the disease, donkeys are considered to be an ideal sentinel species that can be used in the determination of prevalence and distribution of AHSV through the detection of specific antibodies resulting from natural infection [7]. At present, there is no information on the prevalence and distribution of AHSV serotypes in the different administrative as well as foot and mouth disease epidemiological (designated north and south) regions of Namibia.

Therefore, this survey aimed to fill this knowledge gap by investigating the AHSV seroprevalence in Namibian donkeys.

## Materials and Methods

### Ethical approval

The study received ethical clearance from the Animal Research Ethics Committee of the University of Namibia.

### Study area

**Figure-1:**
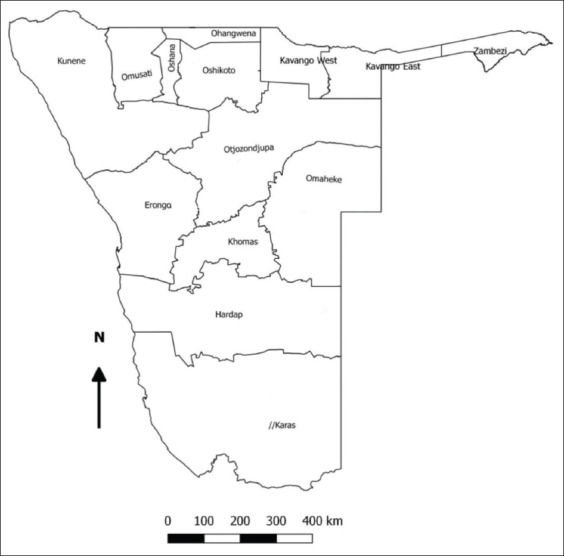
Namibian regions.

Namibia is located at 22°58’1.42”S and 18°29’34.80”E in the southwestern part of Africa. It is divided into 14 administrative regions, as shown in Figure-1. A veterinary cordon fence separates Northern Namibia from the southern country parts. Zambezi, Kavango East, Kavango West, Oshikoto, Ohangwena, Oshana, Omusati, and Kunene are the regions located in the northern of Namibia while Erongo, Otjozondjupa, Omaheke, Khomas, Hardap, and Karas are the Southern regions.

### Samples collection

Between October 2018 and July 2019, blood samples were randomly collected from donkeys by professional veterinarians in 13 administrative regions of Namibian. No samples were collected from the Zambezi region because the region does not have donkeys. A total of 260 blood samples (20 samples for each region) were collected randomly from unvaccinated donkeys aged between 3 and 5 years that had never been out of these regions. The blood was allowed to stand overnight to facilitate clotting. Serum was separated by centrifugation at 3000 rpm for 5 min, refrigerated, and sent to the Central Veterinary Laboratory in Windhoek for AHSV serological screening.

### Serological tests

All the 260 sera were screened for AHSV antibody and viral serotype screening. AHSV-specific immunoglobulin (Ig) G antibodies were detected using a commercial competitive enzyme-linked immunosorbent assay (c-ELISA) kit (Ingezim AHSV, Compact Plus, Spain). To evaluate the AHSV serotype-specific immune response, c-ELISA-positive samples were further tested by SN assay. For the SN test, sera were inactivated at 56°C for 30 min before testing. VERO cells provided by the European Collection of Authenticated Cell Cultures (Public Health England, United Kingdom) were used at a concentration of 100,000 cells/ml for the test. Sera were diluted from 1:10 to 1:1280 and then incubated for 60 min with 100 TCID_50_ of previously titrated AHSV. The virus-serum mixtures were added to 96-well plates with confluent cell monolayers. The specific cytopathic effect (CPE) was evaluated under a light microscope after 5 days of incubation at 37°C in 5% CO_2_. Neutralizing titer was defined as the reciprocal of the highest dilution of serum able to neutralize at least 75% of the virus CPE.

### Statistical analysis

Descriptive and inferential statistics were performed using the Statistical Package for the Social Sciences version 25 (IBM Corp., NY, USA). The Chi-square test was used to test differences in the proportional occurrence of AHS in donkeys from the northern and southern regions as well as from the individual administrative regions of Namibia, whereby p≤0.05 was considered statistically significant.

## Results

**Figure-2:**
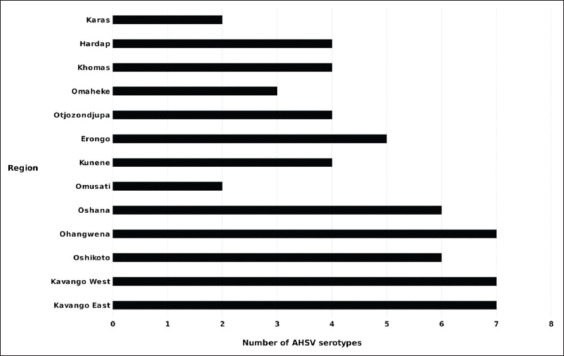
African horse sickness virus serotypes seroconversion found in donkeys by region.

The overall seroprevalence of AHSV-specific IgG antibodies in Namibian donkeys was 63.5% (Table-1). There was, however, a significant difference in the seroprevalence of AHSV-specific antibodies in donkeys from the different administrative regions (p<0.05). Donkeys sampled from Kavango East and Kavango West regions had the highest prevalence of anti-AHSV antibodies (100% and 100%, respectively; p<0.05), whereas those from the Karas region had the lowest AHSV-IgG seroprevalence (40%, p<0.05). As shown in Table-2, the AHSV-IgG seroprevalence was significantly higher in donkeys from the northern regions than in those from the southern regions (64% and 36%, respectively; p<0.05). There was a significant difference in the distribution of AHSV serotype-specific antibodies in donkeys sampled from the different administrative Namibian regions (p<0.05). Overall, significantly higher proportions of donkeys had antibody against AHSV-6 and AHSV-9 (37.8% and 37.8%, respectively; p<0.05). The seroprevalence of AHSV-2 antibodies in donkeys from the northern regions was significantly higher than those from the southern regions (3.9% and 0%; respectively, p<0.05). None of the donkeys in this study, however, tested positive for AHSV-8 serotype-specific antibodies. As shown in Figure-2, the SN assay revealed that the AHSV-IgG-positive donkeys from Kavango East, Kavango West, and Ohangwena regions developed a seroconversion to seven of the nine AHSV serotypes (excluding AHSV-7 and AHSV-8), while AHSV-IgG-positive donkeys from Karas and Omusati regions developed a seroconversion only to AHSV-6 and AHSV-9 serotypes.

**Table-1:** Seroprevalence of AHSV-specific IgG in donkeys according to Namibian regions.

Namibian regions	Number of donkeys positive by AHSV c-ELISA (%)
Northern regions	
Kavango East	20 (100)
Kavango West	20 (100)
Oshikoto	12 (60)
Ohangwena	12 (60)
Oshana	12 (60)
Omusati	14 (70)
Kunene	12 (60)
Southern regions	
Erongo	14 (70)
Otjozondjupa	12 (60)
Omaheke	10 (50)
Khomas	10 (50)
Hardap	9 (45)
Karas	8 (40)
Total	165 (63.5)

AHSV=African horse sickness virus, IgG=Immunoglobulin G, ELISA=Enzyme-linked immunosorbent assay

**Table-2:** Distribution of AHSV serotypes antibodies in donkeys originated from the northern (n=7) and southern regions (n=6) of Namibia.

Serotype	Northern regions (%)	Southern regions (%)	Overall (%)
AHS 1	13 (3.0)	8 (1.8)	21 (4.8)
AHS 2	17 (3.9)	0 (0.0)	17 (3.9)
AHS 3	6 (1.4)	0 (0.0)	6 (1.4)
AHS 4	15 (3.4)	14 (3.2)	29 (6.7)
AHS 5	24 (5.5)	8 (1.8)	32 (7.3)
AHS 6	102 (23.4)	63 (14.4)	165 (37.8)
AHS 7	0 (0.0)	1 (0.2)	1 (0.2)
AHS 9	102 (23.4)	63 (14.4)	165 (37.8)
Total	279 (64.0)	157 (36.0)	436 (100.0)

AHSV=African horse sickness virus, AHS=African horse sickness

## Discussion

Annual polyvalent prophylactic immunization against AHSV is practiced in the majority of horses in Namibia [8]. SN assay is not able to distinguish AHSV serotype-specific antibodies derived from immunization, natural infection, or colostrum [7] and a seroepidemiological survey based on the study of the AHSV seroconversion in horses would have been distorted and unreliable. Infection with AHSV is usually asymptomatic in donkeys. As a result, donkeys do not routinely receive prophylactic immunization against AHSV and are, therefore, ideal candidates for epidemiological AHSV serosurveys to determine AHSV field strains circulating in Namibia.

This survey indicated that the majority of Namibian donkeys (63.5%) had been exposed to AHSV. The results of the current study are in agreement with studies from other African countries that have reported donkey AHSV prevalence of between 59.3% and 72% [9,10]. It would appear that all AHSV serotypes, except AHSV-8, occur in Namibia. More specifically, AHSV-1, -2, -3, -4, -5, -6, and -9 serotypes were identified in donkeys in the wet northern regions of Namibia, while AHSV-1, -4, -5, -6, -7, and -9 were detected in the dry semi-desert areas in the southern part of Namibia.

AHSV serotype 9 is not included in the AHS commercial vaccine available in Southern Africa because it is considered to be of low virulence and antigenically closely related to AHSV-6 [11,12]. Moreover, the development of a fully protective vaccine against all nine AHSV serotypes in the horse population may take many vaccination courses over several years [13,14]. A recent study demonstrated the circulation of AHSV serotypes 1, 2, 4, 6, 7, 8, and 9 in a few Namibian regions with AHSV-9 being responsible for several AHS outbreaks [8]. AHSV-9 and AHSV-6 were the most prevalent serotypes in the donkeys surveyed in this study.

The isolation of AHSV-8 strain in horses and the absence of seroconversion to the AHSV-8 in all the tested donkeys suggest the possibility of AHSV-8 outbreaks in horses linked to reassortment and reversion of the commercial live-attenuated vaccine as previously described [14,15-17]. SN results showed that several donkeys from Kavango East, Kavango West, and Ohangwena regions might have been exposed to multiple serotypes. Because virus reassortment can occur in nature when a single host or vector cell is coinfected by two or more viruses with segmented genome [6,18,19], the use of multiple attenuated strain vaccines in horses and the detection of multiple strains in donkeys indicate the possibility that cocirculation of several strains occurs in Namibia.

## Conclusion

This study highlighted a high level of exposure of donkeys to AHSV in Namibia with the highest prevalence of seroreactors occurring in the northern regions. Except for AHSV-8, all AHSV serotypes have been circulated or are circulating in Namibia and the predominant AHSV serotypes were AHSV-6 and AHSV-9. Due to the wide spatial distribution of AHSV strains circulating in the field, it is recommended that horses in Namibia be vaccinated as per the recommended protocols to prevent the disease.

## Authors’ Contributions

UM designed, coordinated, performed the experiment, and wrote the manuscript. GZ, and SK performed the experiment. EK and GS analyzed the data. BC, IB, CN, and OM collected the samples. BM wrote and edited the manuscript. ND supervised the study. The final manuscript has been read and developed in consultation with all authors. All authors have read and approved the final manuscript.
